# Assessing the Knowledge of the Osteopathic Profession in New York City’s Eastern European Communities

**DOI:** 10.7759/cureus.21664

**Published:** 2022-01-27

**Authors:** Justin Chin, Lina Kleyn, Emily Dube, Mark Terrell, Christine M Lomiguen, Mikhail Volokitin

**Affiliations:** 1 Department of Family Medicine, LifeLong Medical Care, Richmond, USA; 2 Department of Medical Education, Lake Erie College of Osteopathic Medicine, Erie, USA; 3 Department of Family Medicine, Morristown Medical Center, Atlantic Health System, Morristown, USA; 4 Department of Pathology, New York University Grossman School of Medicine, New York, USA; 5 Department of Family Medicine, Millcreek Community Hospital, Erie, USA; 6 Department of Osteopathic Manipulative Medicine, Touro College of Osteopathic Medicine, New York, USA

**Keywords:** low back pain (lbp), russian massage, awareness, recognition, russian, eastern european, osteopathic manipulative treatment (omt), omt, omm, osteopathic manipulative medicine (omm)

## Abstract

Background

According to the decennial Osteopathic Survey of Healthcare in America, the osteopathic profession has been steadily gaining recognition in the United States, particularly among the White/Caucasian demographic. This, however, does not take into account immigrant European communities that, while racially classified as White/Caucasian, may be unexposed to osteopathic physicians (DOs) in their home country and may be reticent to osteopathic manipulative medicine. Data on non-English-speaking communities are limited and can mask the need for further outreach. This study aimed to identify literature in osteopathic outreach to minority communities and assess osteopathic awareness in New York City’s Eastern European communities. Secondary objectives include characterization of potential barriers in hindering access to osteopathic medicine, and, by extension, other minority groups.

Methodology

An anonymous survey prepared in Russian and English was used to gather demographics, education level, healthcare habits, and knowledge of the osteopathic profession. To provide a clinical scenario, a health habit question regarding low back pain (LBP) was provided to participants. Participants over the age of 18 were randomly selected from high density Eastern European areas at two separate time points. Statistical analysis was performed using R to evaluate independence between questions using chi-square tests.

Results

A total of 150 surveys met the inclusion criteria, with 71 males and 79 females, an age range of 18-92, and a median age of 62. On comparing demographics, education level, and healthcare habits, only English proficiency showed statistical significance (p = 0.039) in determining recognition of the osteopathic profession. Overall, 60% (n = 94) stated that they have heard of osteopathic medicine and knew what a DO physician does. However, only 35% (n = 53) would see a DO for LBP, with 50% (n = 77) seeing a physical therapist.

Conclusions

Compared to research examining osteopathic awareness in ethnic minority communities, the Russian community in New York appears to have greater recognition of the osteopathic profession. This, however, does not translate into a clinical scenario as more participants were more likely to see a physical therapist. While this difference can be attributed to numerous factors, it stands without doubt that greater osteopathic outreach and data collection needs to be performed in minority communities.

## Introduction

Osteopathic manipulative medicine (OMM) is a branch of medicine that utilizes manual techniques to diagnose, treat, and prevent illness or injury [1]. Osteopathic physicians (DOs) undergo the same medical education and training in the United States as allopathic physicians (MDs), with the addition of 100+ hours of time dedicated to learning osteopathic principles and osteopathic manipulative treatment (OMT) [2]. International recognition of DOs and OMM, however, has been relatively limited, despite practice privileges in over 50 countries. While DOs have had full practice rights in Russia since 2006, there is little information or no known practice rights in the rest of the former Soviet Union countries [3]. With allopathic physicians serving as the primary healthcare provider in their native countries, many immigrant communities may have never been exposed to DOs prior to re-establishing healthcare in America. Previous studies on osteopathic recognition have delved into this effect in Asian communities and the overall American population, but none have investigated Eastern European or Russian-speaking enclaves [4-8].

This study aims to investigate osteopathic awareness by assessing the familiarity of DOs and OMM in one of the nation’s largest Eastern European diasporas - New York City, New York’s Brooklyn, and Queens boroughs. Greater osteopathic outreach and education are needed in these communities to increase access to primary care providers and alternative care options for chronic pain management [4,5]. This project expanded on previous frameworks on research in minority communities whose primary language is one other than English and further characterized potential barriers that may exist in hindering access or utilization of OMM and, by extension, overall healthcare.

## Materials and methods

**Figure 1:**
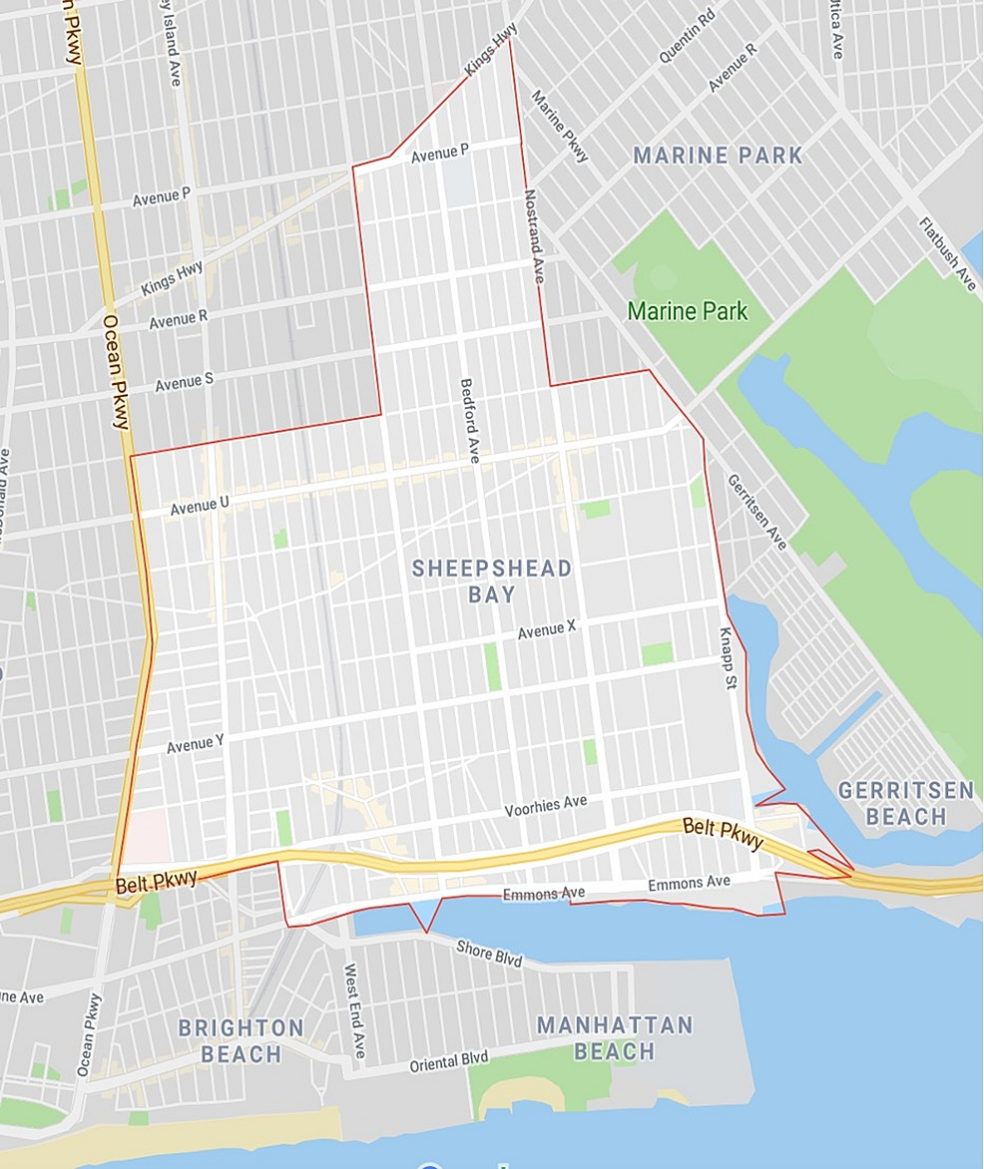
Map of high-density areas in Sheepshead Bay in Brooklyn selected for the desired population. Adjacent to this enclave is Brighton Beach, which also has a high proportion of Eastern European communities. Created on Google Maps using demographic boundaries supplied by census data from New York City [9].

New York has the highest percentage of Eastern European immigrants, accounting for 15% or 2.1 million members nationwide according to the 2010 census [9]. The vast majority reside in New York, New York (New York City) in the boroughs of Brooklyn and Queens in Brighton Beach and Sheepshead Bay. Participants resided in known high-density locations of the Eastern European enclave of Sheepshead Bay in Kings County (Figure 1). Of note, New York City is considered the de facto gateway for Eastern European immigration, with local registries documenting four waves of immigration in the last century, the largest occurring in the 1970s with a mass exodus of Russian-speaking Jews from Soviet Union countries. The current demographics mirror these patterns with pockets of residents hailing from Russia, Ukraine, Belarus, and other former Soviet Union countries in Central Asia, which this study uses as its delineation of Eastern European. 

Participants were informed, both verbally and with the inclusion of a cover letter, that participation was voluntary, and responses required no identifiers to protect the anonymity of participants. Minors, those who did not demonstrate a complete understanding of the basis of the survey, and those who were unable to give informed consent were excluded from this study. This study was approved by the Touro College Health Sciences Institutional Review Board for the Protection of Human Subjects (HSIRB #1795E).

Measures

**Figure 2:**
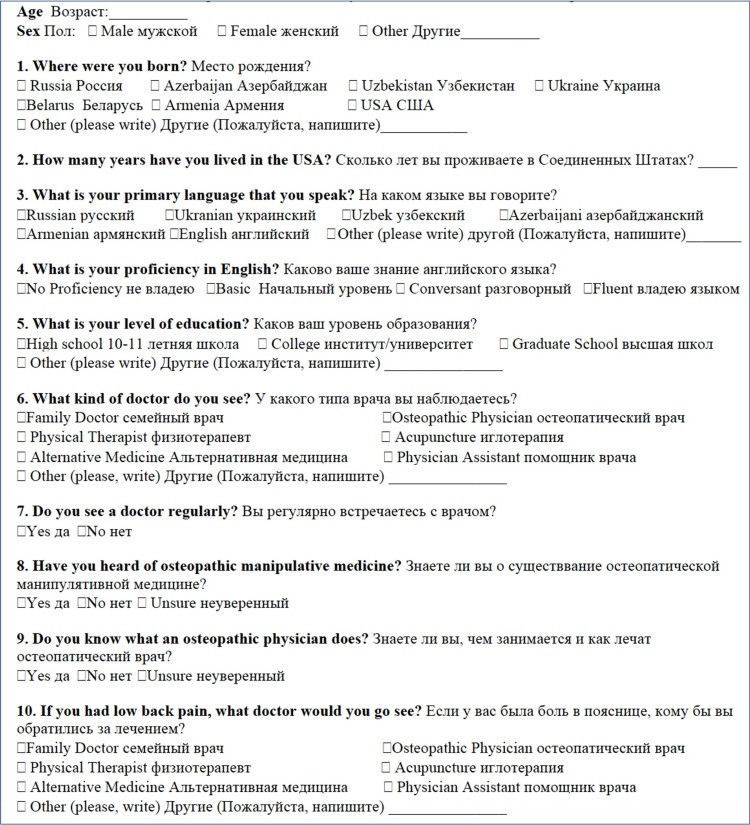
Survey questions in Russian and English. Russian survey adapted by Chin et al. from prior surveys on osteopathic recognition in ethnic minorities [4-6]. This survey has been validated through Institutional Review Board review and reviewed in Russian by a physician at Touro College of Osteopathic Medicine who actively practices osteopathic manipulative medicine and is a Diplomate of the National Board of Osteopathic Medical Examiners in Medicine and Surgery.

A 12-question, mixed, multiple-choice, and dichotomous (yes/no) survey was developed specifically for this study to measure osteopathic awareness. The survey was provided on paper in English and Russian (Figure 2). It included questions regarding demographics (age, gender, education level), language (primary language, English proficiency), healthcare habits (regularity of doctor visits, type of doctors visited), knowledge of OMM, and a clinical scenario of low back pain (LBP), one of the most common reasons for doctor visits and one for which OMT has been shown to effectively treat, was provided to participants.

Data collection and analysis

Medical student researchers were deployed within the municipal delineations for the Sheepshead Bay neighborhood in the New York City borough of Brooklyn and utilized convenience sampling in high-density areas, including major thoroughfares and parks, to identify participants available for the study. All subjects were invited to participate as no inclusion criteria were identified prior to sampling. Participation was strictly voluntary as no specific recruitment methodologies were used and no financial reimbursement or other compensation was provided. Collection occurred over four consecutive days on two separate occasions, October 18 through October 21, 2018, and October 25 through October 28, 2018, totaling eight days.

Survey data were scanned, and a data spreadsheet was electronically created using a licensed version of Microsoft Excel, version 2016 (Microsoft Corporation, Redmond, WA, USA). The data were subsequently coded for statistical analysis. Group comparisons were completed using Pearson’s chi-square tests (χ^2^ tests) of independence to examine the difference, if any, between health habits and demographics (age, sex, birth location, years in the United States, primary language, English proficiency, and education level) and awareness of the DO profession and knowledge of OMM. Statistical analysis was performed using the release version R-2.15.3.tar.gz of R: A Language and Environment for Statistical Computing, developed in Vienna, Austria by the Core Team of the Foundation for Statistical Computing [10].

## Results

A total of 150 participants were surveyed and included in the final analyses of participant demographics versus recognition of DOs and OMM. In total, 71 males and 79 females were included in the study, with an age range of 18-92 and a mean age of 68 ± 8.96. Of the 150 participants surveyed, only 40% (n = 60) indicated knowledge about DOs; however, 62% (n = 93) demonstrated knowledge of OMM, with a good distribution of demographic qualities spread across the community. Detailed demographic data and results with statistically significant factors are displayed in Table 1.

**Table 1:** Demographic characteristics of all participants compared with participants with knowledge of DOs and OMM. DO: doctor of osteopathic medicine; OMM: osteopathic manipulative medicine

Characteristic	All participants (n = 150)	Knowledge of DOs (n = 60)	Without knowledge of DOs (n = 90)	P-value	Knowledge of OMM (n = 93)	Without knowledge of OMM (n = 57)	P-value
Sex							
Male	71 (44.76%)	28 (46.67%)	43 (47.78%)	0.9765	49 (52.69%)	22 (38.60%)	0.2428
Female	79 (55.24%)	32 (53.33%)	47 (52.22%)		44 (47.31%)	35 (61.40%)	
Age (years)							
Median	68	66	67	0.6258	66	67	0.2142
18–29	28 (18.67%)	11 (18.34%)	17 (18.89%)		21 (22.58%)	7 (12.28%)	
30–39	15 (10.00%)	5 (8.33%)	10 (11.11%)		10 (10.75%)	5 (8.77%)	
40–49	6 (4.00%)	2 (3.33%)	4 (4,44%)		5 (5.38%)	1 (1.76%)	
50–59	14 (9.33%)	8 (13.33%)	6 (6.67%)		12 (12.90%)	2 (3.51%)	
60–69	19 (12.67%)	6 (10.00%)	13 (14.44%)		12 (12.90%)	7 (12.28%)	
70–79	29 (19.33%)	9 (15.00%)	20 (22.22%)		12 (12.90%)	17 (29.82%)	
80–90	28 (18.67%)	15 (25.00%)	13 (14.44%)		15 (16.14%)	13 (22.81%)	
>90	11 (7.33%)	4 (6.67%)	9 (10.00%)		6 (6.45%)	5 (8.77%)	
Location of birth							
Russia	27 (18.00%)	12 (20.00%)	15 (16.67%)	0.9542	16 (17.20%)	11 (19.30%)	0.2404
Azerbaijan	10 (6.67%)	4 (6.67%)	6 (6.67%)		6 (6.45%)	4 (7.02%)	
Uzbekistan	14 (9.33%)	3 (5.00%)	11 (12.21%)		4 (4.30%)	10 (17.54%)	
Ukraine	49 (32.67%)	18 (30.00%)	31 (34.44%)		30 (32.26%)	19 (33.33%)	
Belarus	28 (18.67%)	12 (20.00%)	16 (17.78%)		22 (23.66%)	6 (10.53%)	
United States	8 (5.33%)	3 (5.00%)	5 (5.56%)		5 (5.38%)	3 (5.26%)	
Other	14 (9.33%)	8 (8.33%)	6 (6.67%)		10 (10.75%)	4 (7.02%)	
Length of time in the United States (years)							
0–5	3 (2.00%)	1 (1.67%)	2 (2.22%)	0.7913	2 (2.15%)	1 (1.75%)	0.3507
6–10	3 (6.00%)	1 (1.67%)	2 (2.22%)		2 (2.15%)	1 (1.75%)	
11–15	10 (6.67%)	4 (6.67%)	6 (6.67%)		7 (7.53%)	3 (5.26%)	
16–20	35 (23.33%)	15 (25.00%)	20 (22.22%)		21 (22.58%)	14 (24.56%)	
21–25	57 (38.00%)	23 (38.33%)	34 (37.78%)		30 (32.26%)	27 (47.38%)	
>26	42 (28.00%)	16 (26.66%)	26 (28.89%)		31 (33.33%)	11 (19.30%)	
Highest level of education							
Elementary	24 (16.00%)	11 (18.33%)	13 (14.44%)	0.0931	12 (12.90%)	12 (21.05%)	0.4473
High school	101 (67.33%)	39 (65.00%)	62 (68.90%)		63 (67.74%)	38 (66.67%)	
College	22 (14.67%)	10 (16.67%)	12 (13.33%)		17 (18.28%)	5 (8.77%)	
Graduate School	3 (2.00%)	0	3 (3.33%)		1 (1.08%)	2 (3.51%)	
English proficiency							
No proficiency	13 (8.67%)	5 (8.33%)	8 (8.89%)	0.7612	5 (5.38%)	8 (14.04%)	0.0396*
Yes proficiency	137 (91.33%)	55 (91.67%)	82 (91.11%)		88 (94.62%)	49 (85.96%)	
Basic	45 (32.85%)	16 (29.09%)	29 (35.37%)		24 (27.27%)	21 (42.86%)	
Conversational	39 (28.47%)	16 (29.09%)	23 (28.05%)		25 (28.41%)	14 (28.57%)	
Fluent	53 (38.68%)	23 (41.82%)	30 (36.58%)		39 (44.32%)	14 (28.57%)	
Primary language							
English	30 (20.00%)	10 (16.67%)	20 (22.22%)	0.8161	21 (22.58%)	9 (15.79%)	0.0075*
Not English	120 (80.00%)	50 (83.33%)	70 (77.78%)		72 (77.42%)	48 (84.21%)	
Russian	110 (91.67%)	47 (94.00%)	63 (90.00%)		67 (93.06%)	43 (89.58%)	
Ukrainian	6 (5.00%)	2 (4.00%)	4 (5.71%)		4 (5.55%)	2 (4.17%)	
Uzbek	2 (1.66%)	0	2 (2.86%)		0	2 (4.17%)	
Other	2 (1.66%)	1 (2.00%)	1 (1.43%)		1 (1.39%)	1 (2.08%)	

In our study, knowledge of DOs and OMM was the highest among English-proficient participants, with a scattered distribution found in other demographics. The primary language spoken at home and English fluency were the sole statistically significant factors for whether participants had knowledge of DOs and OMM. Russian as the primary language spoken at home and English proficiency were statistically significant (p ≤ 0.05, p = 0.0075, and p = 0.0396, respectively, Table 1). Among the Eastern European community members surveyed, no significant differences in knowledge of DOs or OMM were present among groups when stratified based on sex, age, location of birth, number of years living in the United States, and level of education completed (Table 1).

Concerning the healthcare habits of the study participants, no significant difference in knowledge of DOs or OMM was found between those who visited their doctor regularly versus those who did not see their doctor regularly (Table 2). Of those participants who see their doctor regularly, 87% reported seeing their family physician (Table 2). Concerning the clinical scenario of LBP that was presented to the study participants, those who had knowledge of DOs and OMM stated they would see a DO; however, the majority elected to see a physical therapist (Table 2).

**Table 2:** The health habits of participants versus those with knowledge of DOs and OMM. DO: doctor of osteopathic medicine; OMM: osteopathic manipulative medicine; LBP: low back pain

Question	All participants (N = 150)	Knowledge of DOs (n = 60)	Without knowledge of DOs (n = 90)	P-value	Knowledge of OMM (n = 93)	Without knowledge of OMM (n = 57)	P-value
Do you see a doctor regularly?							
Yes	119 (79.33%)	49 (81.67%)	70 (77.78%)	0.8404	73 (78.49%)	46 (80.70%)	0.9460
No	31 (20.67%)	11 (18.33%)	20 (22.22%)		20 (21.51%)	11 (19.30%)	
What kind of doctor do you see?							
Family doctor	130 (86.66%)	51 (85.00%)	79 (%)	0.3680	81 (87.10%)	49 (85.96%)	0.1195
OMM physician	4 (2.67%)	4 (6.67%)	0		4 (4.30%)	0	
Physical therapy	13 (8.67%)	4 (6.67%)	9 (%)		7 (7.53%)	6 (10.53%)	
Other	3 (2.00%)	1 (1.66%)	2 (%)		1 (1.07%)	2 (3.51%)	
With LBP, what doctor would you see?							
Family doctor	53 (35.34%)	22 (36.67%)	31(34.45%)	0.9893	34 (36.56%)	19 (33.33%)	0.1241
OMM physician	14 (9.33%)	10 (16.67%)	4 (4.44%)		12 (12.90%)	2 (3.51%)	
Physical therapy	77 (51.33%)	26 (43.33%)	51 (56.67%)		43 (46.24%)	34 (59.65%)	
Other	6 (4.00%)	2 (3.33%)	4 (4.44%)		4 (4.30%)	2 (3.51%)	

## Discussion

Among survey participants, there is a general awareness of DOs and OMM in the Eastern European-Russian community in New York City’s Sheepshead Bay community. The primary language spoken at home (English) and English proficiency (self-reported level) were the sole statistically significant factors found in the knowledge of DOs and OMM. Compared to similar studies conducted in Eastern and Southern Asian populations, this inaugural study on the Eastern European community showed higher levels of DO and OMM recognition. The decennial Osteopathic Survey of HealthCare in America (OSTEOSURV) is the sole prognosticator for osteopathic recognition in the United States; however, it tends to generalize findings under larger racial categories [7,11,12]. As delineated by the American Osteopathic Association (AOA), Eastern European is considered under the “White/Caucasian” umbrella which follows guidelines set by the United States National Library of Medicine [13]. In comparing this study with previous OSTEOSURV results, there are comparable levels of DO and OMM knowledge; however, much higher rates were found in OSTEOSURV, which may be due to OSTEOSURV respondents primarily based in the United States Midwest, an area that traditionally has high osteopathic recognition due to the historical roots of the osteopathic profession [14]. Therefore, this study was necessary to shed light on perspectives within the White racial monolith and determine whether further outreach is needed based on ethnocultural or geographical differences.

Compared to its long history in the United States, DOs and OMM are relatively new in Eastern Europe and Russia as state-sanctioned and regulated osteopathic medical schools did not develop until the early 2000s [15]. The Russian Higher School of Osteopathic Medicine was the first osteopathic medical school in 1996, and prior to this, osteopathic practitioners were largely unregulated as they were primarily MDs who obtained additional osteopathic training through various professional development programs or fellowships [16]. According to the AOA, Russian recognition of DOs did not occur until 2006, and non-Russian DOs are still required to obtain sponsorship through local hospitals or facilities [17]. The role of American DOs and their practice privileges are unclear in other Eastern European countries and likely require individual inquiry and discussion with the relevant medical board of that country. International licensure and practice rights continue to be a priority for the AOA; however, it does not explain the relative familiarity of New York City’s Eastern European community with the osteopathic profession [18]. Compared to other similar studies, however, this community tended to have a greater length of years in the United States but was not statistically significant.

Manual manipulation of the musculoskeletal system, however, is not unknown to the Eastern European/Russian population. Formulated in the late 18th century, massage therapy was utilized extensively in the Eastern European medical field because it was considered a component of physical rehabilitation and therapy [19]. Often cited as the father of Russian massage, Dr. M.Y. Mudrov was an equivalent contemporary to Dr. A.T. Still for osteopathic medicine [20,21]. Russian massage therapy consists of light, superficial strokes combined with pressure through the finger pads. A primary difference, however, was that since its inception, Russian massage was incorporated into standard medical care rather than a unique or separate branch [21]. Exposure to Russian medical massage tended to occur at an early age, with infant massages developed in the late 19th century as a way to increase blood circulation, which, in turn, was believed to promote enhanced child physical and mental development [22]. Similarly, osteopathic craniosacral and effleurage techniques have also been performed in infancy and childhood with similar success [23,24]. Massage after obstetric delivery, surgical procedures, and sports injuries were developed in the Soviet Union during the same period that osteopathic research for similar indications was being developed [25]. It is this similarity that may play a role in the relative acceptance of osteopathic medicine in the Russian/Eastern European community.

New York Institute of Technology College of Osteopathic Medicine (NYITCOM’s) Émigré Physicians Program (EPP) presents a potentially unique rationale for the relative osteopathic recognition in New York’s Eastern European/Russian community [26]. Established in the early 2000s, NYITCOM’s EPP retrains physicians born and educated outside the United States in obtaining a DO degree. The EPP curriculum blends osteopathic education with clinical experiences and has graduated over 200 physicians in the past two decades, with 30% of participants coming from Russia or former Soviet Union countries [27]. Family medicine and other primary care specialties comprise the majority of residencies for EPP graduates, with greater than 50% ultimately staying in New York [28]. Subsequent canvassing of the study area revealed a sizable proportion of Russian/Eastern European physicians in the Sheepshead Bay community, some of whom were from the NYITCOM EPP program, approximately 30 miles apart. The observance of ethnic enclaves in combination with Russian-speaking osteopathic physicians may play a role in the higher than expected recognition of the osteopathic profession because word-of-mouth and physician referrals can play a role in greater osteopathic uptake [29]. In the clinical scenarios, however, a physical therapist was more likely to be referred to in LBP, which may reflect insurance authorization practices versus a lack of osteopathic referrals [30]. Therefore, a multilayered approach and contextual/nuanced view are needed if the relative success of osteopathic awareness and recognition is to be replicated in communities that lack exposure to the field.

Limitations exist with this study. While New York City has concentrated areas of Eastern European immigrants, convenience sampling in one area, specifically the Sheepshead Bay neighborhood in the borough of Brooklyn, may not provide a true representation of osteopathic awareness, along with contributing to a low sample size. Additionally, there is the possibility that survey participants were unaware that a family doctor could be either an MD or DO. Further studies could sample other areas of Brooklyn, as well as other boroughs of New York City. Moreover, the survey could be modified to assess awareness and perception of family doctors as either MDs or DOs.

## Conclusions

The results of this study provide evidence of the general awareness of DOs and OMM in the Eastern European-Russian community in the Sheepshead Bay neighborhood of the borough of Brooklyn in New York, NY, USA. The statistically significant factors contributing to this awareness include (1) English as the primary language spoken at home and (2) self-reported English proficiency. Compared to similar studies done in Eastern and Southern Asian populations, this inaugural study on the Eastern European community showed higher levels of DO and OMM recognition. A contributing factor to this recognition may be the nearby osteopathic medical school special program for physician immigrants, with 30% of participants from Russia or former Soviet Union states, with greater than 50% graduating and remaining in New York to practice. Similar programs can be established by other osteopathic medical schools to increase cultural awareness of DOs and OMM. This study is limited by sample size and convenience sampling in one neighborhood. However, while 87% of participants admitted to seeing a family doctor (versus less than 3% seeing a DO), with 35% of participants indicating they would see a family doctor for LBP (versus 9% who would see a DO), the survey did not indicate a family doctor could be either an MD or DO. Modification of this survey may result in higher awareness of DOs and OMM in the Eastern European community than the results of this study reveal. This study encourages researchers to design similar studies to assess other ethnic minority communities and their knowledge of the osteopathic field, with contributing and limiting factors to broaden outreach efforts.
